# Supplementary material for: The incremental value of the contribution of a biostatistician to the reporting quality in health research—A retrospective, single center, observational cohort study
Source: PLoS One. 2022 Mar 4;17(3):e0264819. doi: 10.1371/journal.pone.0264819 (PMC8896706; doi:10.1371/journal.pone.0264819)
Supplement: S1 Appendix — Search string for the identification of potential control publications. (PDF) [file pone.0264819.s001.pdf]

Appendix 1. Search string

Year 2017
("2017.01.01"[Date - Publication] : "2017.12.31"[Date - Publication]) AND medline[sb] AND "english"[Language] AND hasabstract AND ("zurich"[Affiliation] OR "zuerich"[Affiliation] OR "zürich"[Affiliation]) NOT ("1900.01.01"[Date - Publication] : "2016.12.31"[Date - Publication])

Year 2018
("2018.01.01"[Date - Publication] : "2018.12.31"[Date - Publication]) AND medline[sb] AND "english"[Language] AND hasabstract AND ("zurich"[Affiliation] OR "zuerich"[Affiliation] OR "zürich"[Affiliation]) NOT ("1900.01.01"[Date - Publication] : "2017.12.31"[Date - Publication])

Date when controls on PubMed were assessed + list of all PMIDs: 2019-12-16
Date when cases on PubMed were assessed: 2019-12-09
