# Supplementary material for: The incremental value of the contribution of a biostatistician to the reporting quality in health research—A retrospective, single center, observational cohort study
Source: PLoS One. 2022 Mar 4;17(3):e0264819. doi: 10.1371/journal.pone.0264819 (PMC8896706; doi:10.1371/journal.pone.0264819)
Supplement: Attachment — Submitted filename: PONEreview_notes.docx [file pone.0264819.s004.docx]

**Synopsis of detailed Draft/Publication Review**

**Descriptors**

**Identifier**: PONE-D-21-37986.

**Title**: “The incremental value of the contribution of a biostatistician to the reporting quality in health research - a retrospective, single center, observational cohort study” [BIQMR]

**Corresponding Author**: Ulrike Held, PhD, University of Zurich: Universitat Zurich, Zurich, SWITZERLAND

“Our study is the first to demonstrate a higher reporting quality and methodological strength in health research publications with a biostatistician on the author list. The higher reporting quality persisted through subgroups of study types and dimensions. Methodological knowledge gaps were identified for prediction / prognostic studies, and for reporting on statistical methods in general and missing values, specifically.”

**Dates**: 12/19/2021-12/26/2021

**Summary of Review**

*Publication recommended, with:*

--expansion of or expounding/clarification of bias sections notes [see lines 145-156]

--R Studio resource link: i.e. <https://shiny.rstudio.com/> or better—[add to test or appendix].

--provide a “visualization” example, flowchart, something for exemplification [see lines 163-165]

--“Table 1. Descriptive Statistics”, a footnote detailing the “other” software products might be worth considering (although not required). [after line 203]

*Optionals (for readers):*

--Discussion Section. further description, definition, examples of “sub optimal reporting” [254-256]; are there qualifiers defined for this? —[add to appendix]

--summary of all qualifiers/quantifiers for 0,1,2 scoring, as used by raters—[add to appendix].

--rater/review sheet, etc. [preferred, although optional(?)] actual sample of result, or idealized example of result, in format used to keep the notes and observations, either onscreen, in a (excel like) table, on paper, in a notebook, etc.. —[add to appendix]

--data descriptive metadata file—[add to appendix]

**Review Notes**

The objectives of the study: i) “quantify the contribution of a biostatistician as co-author on the quality of reporting and methodological strength in health research publications”, ii) “identify the dimensions of reporting quality and study types with methodological knowledge gaps”; and iii) “promote the awareness of the importance of good reporting among clinical researchers and biostatisticians”.

A scoring process with a range of 0 through 11 was assigned to each of the objectives under evaluation (six metrics, possible scores for each: 0,1,2). Thirteen known statisticians, recognized by the University of Zurich system, were identified and their inclusion in the authors list was required for the article review.

The assumption made by researchers is that this inclusion in the author’s list implies considerably more engagement in the research and writing processes than serving as mostly a numbers analyst. For this study, these individuals were identified as “exposed”, implying the research team has included the skills and benefits of a ‘full-fledged, focused biostatistician.’

The counter group for this study is the “non-exposed” group, Teams who produced publications of similar features and matching methodologies, using a research team that is absent an official “biostatistician.” Presumably, the individuals engaged in this work, and any others part of the research process they are involved with, engage in biostatistician-related processes, but without the educational background and professional experience typically required for a biostatistician position. Important to note, the authors of this article recognize that some biostatisticians classified as “non-exposed” might in fact be involved in a limited sense, which because of its nature, were not included in the publication’s co- authors list, suggesting as well that a lesser contribution was made by this individual.

As for the publications reviewed, a total of 131 articles were identified for the years 2017 and 2018 that consisted of the predefined 14 biostatisticians employed by University of Zurich and affiliates. Of the 131 articles, the majority of them (95) could be identified as relying upon three specific study types-- Randomized control trials (RCTs), observational studies, and prediction/prognostic. These 95 were matched by a similar sample drawn from a pool of 400, extracted out of the original 2,459 articles identified as having possible institutional connection(s) and meeting the requirements for this study.

In the end, the three study types identified in the “exposed” group for this research – Randomized control trials (RCTs), observational studies, and prediction/prognostic— matched to similar articles contained in the “non-exposed” articles. A number of features related to researchers’ profiles, patients selection processes, data quality, content processes, analytic methods and software utilization, were then applied to identify the ‘best fits’ for each “exposed”-“non-exposed” study pair.

The method used to evaluate and score the qualities of these two pairs of 95 articles underwent similar, multiple research development processes. To develop the metrics for calculating each of these articles, three types of meta-analysis techniques or recommended research were used, to develop a hybrid model of these analytic approaches: ‘Consolidated Standards of Reporting Trials’ (CONSORT), ‘Strengthening the Reporting of Observational Studies in Epidemiology’ (STROBE), and ‘Transparent Reporting of a multivariable prediction model for Individual Prognosis Or Diagnosis’ (TRIPOD). The algorithms developed for this methodology (initial eligible publications query, location/agency related eligibility terminology, and table summarizing the scoring algorithm from each of the three ‘study type’ descriptions just detailed (not reviewed in detail here), are provided in the three appendices attached to this article under review.

Most appropriately, the interrater training required to assure consistent interrater reliability was described in detail. Fifteen raters were recruited, each receiving at least one set of 11-12 publications to review; those who engaged in 33 or more reviews are included in the author list for this article to be published. These ratings took place over a three-month period, and all independent reviews had to be duplicated at least once by different raters.

How much this article selection process was evaluated for bias is uncertain, and the attention paid to bias concerns is briefly described, and is perhaps in need of **slight expansion** [lines 145-156].

Still, the nature by which results were compared internally, between reviewers or raters, seems more than adequate for this study. Some of the details of how this was accomplished are provided. (Perhaps add an **rater/review notes or form/sheet**),

However, a **“metadata” table**, with detailed datum descriptors and perhaps structured and non-structured data forms and examples, and/or a full example of the data gathering form, page or table (mock and hybridized to show complexity of responses), would be a valuable addition to the appendix of this research article.

According to the article, an R Studio/tm Shiny (https://shiny.rstudio.com/) app was employed to assess and test data production, for consistency and reliability. Analyses were performed using R 4.1.1. [lines 180-187] Most of the final reporting of results was based upon STROBE guidelines. (Perhaps there is a set of articles or **a resource link** that could be referenced here and/or added to the appendix regarding this.)

In the ‘Statistical Methods and Programming’ section, a number of graphics are mentioned. A “biostatistician” reading this article would probably appreciate seeing one or more examples of these. Due to the wording—"**visualization** of the results with dot-plots (lollipop plots), in which the means of the outcome in the exposed and non-exposed publications are shown” [lines 163-165]—*as a reader,* *I am expecting at least one example of these for my review.*

The reporting of results is very succinct. I appreciate the breadth of its content.

In “Table 1. Descriptive Statistics”, a **footnote** detailing the “other” software products might be worth considering (although not required). Reason: A limitation of the value of this article to readers, especially those within health care system, is that it may not cater as well to those working only with non-R programming requirements. *What* ***methods and software*** *did the non-R-programmers use*? This could relate to certain groups’ level of involvement with this type of work.

For the “Agreement” and three subsequent sections, results are reported for “agreement” between raters, presentation of primary outcome differences between “exposed” and “non-exposed” groups, and evaluation of for each of the five “dimension-specific” scores (scoring range 0-2, for 5 ‘dimensions—my related research question (RQ): *what were the score determinants for each?* [**appendix**?]’).

Numbers of citations per publication were then documented and evaluated. For the related data (Table 2. Descriptive statistics for number of citations. Estimates show the median [IQR]), the differences between the median and IQR for the two groups requires further contemplation. (RQ: What does a highly detailed and/or lengthy reference bibliography suggest, regarding the comparability of these two studies and their researchers?) I would consider this more as a question to explore in a follow up, for continuing the use for this evaluation method, rather than a change needed for this draft article.

In order to apply, for replication of this work, I still **need to know** and see examples for: “the “Missing data” and “Statistical methods” dimensions . . . generally reported with less detail” (i.e. by word count? Applicability? Nouns? Subjects? ‘breadth’ in some form?) [line 226-228].

Regarding the ‘Discussion’ section, results of this study are very helpful. The author(s) suggest “exposed” publications results demonstrate better subgroups analyses than “non-exposed” publications. The authors also state: “Methodological knowledge gaps seemed to be more prominent in the areas of statistical methods, and missing values.”, suggesting a major attribute of engaging the biostatistician in the work is avoiding “missed opportunities” [252-253] to engage in more complete and accurate reporting, that adequately reviews all potential outcomes related dimensions of the research work.

Another advantage of including biostatisticians, as noted by the authors, is: “Low citation numbers of corresponding reporting guidelines in both, the group of exposed and non-exposed publications may be an indication of lack of awareness among study authors.” [261-263]. The latter is very much confirmed by the various recommendations or scoring guidelines published about CONCERT, STROBE, TRIPOD, AMSTAR, PRISMA, and other meta-analytic scoring processes.

A brief criticism stated by the author(s) is that the biostatisticians’ reporting may still be “sub optimal reporting” [256] in terms of details, inferred by: “While it seems reasonable to assume that in the exposed papers the biostatisticians knew the methods well, there was still sub optimal reporting of these.” [254-256]. This needs **further explanation**.

The “Limitations and Strengths” discussion is useful. ‘Reporting quality’ and ‘methodological strength’ are the main topics reviewed in this section.

To better understand ‘methodological strength’, the **details of the methodologies** have to be provided. **But what about barriers?:** too frequently this part of a publishable research report is provided with inadequate writing space (page/section limitations), by the primary author (lack of awareness), or publisher (publication requirements and guidelines), if not both.

It is also not unusual for studies to be partially limited by the selection process, and the impact of “missing values” (treated as exclusion instead) on the statistical modeling and outcomes. Overly restrictive regression models can exclude too many sample cases, and bias the results presented. Reductions in sample size also impact the indicators of statistical significance, bringing the total N for a study below the “safe”, valid, reliable range of possible participants. (Note: The available sample size for the “non-exposed” group was met (400), and therefore the quality of the 95 that were selected is the only issue; the bibliography of each at first appears to be a mismatch, due to the median-IQR relationships.)

A major benefit of this article is that results demonstrate the value of incorporating fully representative biostatisticians within a given research team. In work settings where this is typical for a research team, group, department, or program to hire a “biostatistician,” their impacts upon institutional projects can be quite noticeable, and arguable, through internal studies of publication rates. However, it seems such internal measures are unusual for institutions. When biostatisticians are included as co-research designers, developers, analysts, and writers, it is likely that rates of publication are increased following first submission, thereby reducing the rates of required rewrites, re-evaluations, data reruns, project extensions, IRB updates, and grant renewals.

Unfortunately, few if any institutions engage in some form of comparative performance measure for research groups. A most recent review performed by a New York City citywide (2M patients) program, of more than 400 projects, engaged in for seven years, revealed standard publication times for projects published and overseen or assisted with by a central office biostatistician, were 3.5 to 4.5 years, whereas published outcomes for projects initiated by researcher and/or central IT developed data pulls, without engaging in a central office biostatistician, were 4.5 to 7 years. Each of the latter required at least one revamp of the research project, including residency staff changes, rewrite, resubmission and re-approval by IRB, per calandar year.

Held *et al*.’s article suggests that role of the epidemiologic biostatistician in research is still not fully understood.

In terms of hiring purposes, when teams utilize internal statisticians (dual degree colleagues) as substitutes, they reduce the potential of what may be discovered or accomplished with their study. This error, in turn, may lesson chances for publication or grant renewal, or lead to ‘incomplete’ or ‘partial’ testing.

For the next stage in this research process, a more complete review needs to be made of the methodologies and approaches to analyses discussed by this article.

Like many dissertations or theses projects, significantly detailed descriptions of the research project must be provided in the final report, including whatever attachments and appendices are needed to allow for further testing, or to help researchers improve upon their methods, formulas, and understanding of math theory, ultimately, to make their results more confirmable and more visible.
